# Nano-scaled polyacrylonitrile for industrialization of nanofibers with photoluminescence and microbicide performance

**DOI:** 10.1038/s41598-024-58035-5

**Published:** 2024-04-04

**Authors:** Hossam E. Emam, Tamer Hamouda, El-Amir M. Emam, Osama M. Darwesh, Hanan B. Ahmed

**Affiliations:** 1 Department of Pretreatment and Finishing of Cellulosic Based Textiles, Textile Research and Technology Institute, National Research Centre, Scopus Affiliation ID 60014618, 33 EL Buhouth St., Dokki, Giza, 12622 Egypt; 2 Spinning and Weaving Engineering Department, Textile Research and Technology Institute, National Research Centre, 33 EL Buhouth St., Dokki, Giza, 12622 Egypt; 3 Faculty of Applied Arts, Textile Printing, Dyeing and Finishing Department, Helwan University, Cairo, 11795 Egypt; 4 Agricultural Microbiology Department, National Research Centre, Giza, 12622 Egypt; 5 Chemistry Department, Faculty of Science, Helwan University, Ain-Helwan, Cairo, 11795 Egypt

**Keywords:** PAN, Nanopolymer, Carbon nanofiber, Thermal treatment, Photoluminescence, Antimicrobial performance, Green chemistry, Nanoscale materials

## Abstract

Nanofibers are investigated to be superiorly applicable in different purposes such as drug delivery systems, air filters, wound dressing, water filters, and tissue engineering. Herein, polyacrylonitrile (PAN) is thermally treated for autocatalytic cyclization, to give optically active PAN-nanopolymer, which is subsequently applicable for preparation of nanofibers through solution blow spinning. Whereas, solution blow spinning is identified as a process for production of nanofibers characterized with high porosity and large surface area from a minimum amounts of polymer solution. The as-prepared nanofibers were shown with excellent photoluminescence and microbicide performance. According to rheological properties, to obtain spinnable PAN-nanopolymer, PAN (12.5–15% wt/vol, honey like solution, 678–834 mPa s), thermal treatment for 2–4 h must be performed, whereas, time prolongation resulted in PAN-nanopolymer gelling or rubbering. Size distribution of PAN-nanopolymer (12.5% wt/vol) is estimated (68.8 ± 22.2 nm), to reflect its compatibility for the production of carbon nanofibers with size distribution of 300–400 nm. Spectral mapping data for the photoluminescent emission showed that, PAN-nanopolymer were exhibited with two intense peaks at 498 nm and 545 nm, to affirm their superiority for production of fluorescent nanofibers. The microbial reduction % was estimated for carbon nanofibers prepared from PAN-nanopolymer (12.5% wt/vol) to be 61.5%, 71.4% and 81.9%, against *S. aureus*, *E. coli* and *C. albicans*, respectively. So, the prepared florescent carbon nanofibers can be potentially applicable in anti-infective therapy.

## Introduction

One-dimensional (1D) nanostructured materials were considerably interested, attributing to their excellent performance and wide-scaled applicability in different fields, such as drug delivery systems, optoelectronic systems, food production, filtration systems, sensing, catalyzing, and scaffolds for the tissue engineering^1–3^. Fiber spinning is ascribed as a versatile and simple technique for generation of 1D nanofibers from an abundant different precursor like synthetic and natural polymers, chromophore containing polymers, polymer alloys and ceramics^4–6^.

Manufacturing of nanofibers as one-dimensional structure could be ascribed as a considerable field of study as they exhibit different advantages^7^, to make it superiorly applicable in different purposes, such as in supercapacitor electrodes^8–10^, drug delivery systems^7,11^, air filters^12^, wound dressing^13–15^, water filters^16^, and tissue engineering^17^. Some synthetic techniques for manufacturing nanofibers are designing, electrospinning, phase separation, self-assembly, and figuring the synthesis^9,10^. However, electrospinning is more applicable for manufacturing of fibers with nano-sized up to micrometers dimensions^7^, attributing to its compatibility to give large-scale nanofibers of the same size from different precursors, in addition to the immense ratio of surface area/volume, to make electrospinning described as a fast and simple, technique^18^. Fiber topography may be controlled by adjusting the experimental parameters of electrospinning process, such as the solution viscosity, conductivity, and surface tension; the voltage (high), flow rate, and needle tip distance to the collector; and environmental humidity^11,19^. Electrospinning can also be applied for effective preparation of fibrous membrane that exhibit active laccase encapsulated inside and surficial nano-scaled channels, to be exploited for direct sorption of organic or inorganic water pollutants, due to their porous structures, high surface area, inter-connectivity, and excellent mechanical characters^20,21^. However, solution blowing fiber spinning as more advantageous process compared to electrospinning, could be identified as a process for production of non-woven fiber sheets from a minimum amounts of polymer solution, whereas, the produced fibers are exhibited with high porosity and an extremely large surface area^22^, compared to that obtained from electrospinning process.

PVA (Polyvinylalcohol), PVP (polyvinylpyrrolidone), CA (cellulose acetate), and PAN (polyacrylonitrile) are synthetic polymer that could be successfully exploited in manufacturing of nanofibers^7,9,11^. However, PAN is preferable for its ability to maintain geometry in the pyrolysis, good solvent resistance, low density, high polymer strength and elasticity^23^, so PAN nanofiber is widely applicable for production of membrane air filters, carbon fibers, and water filters^23,24^. Few reports were considered with preparation of photoluminescent/biologically active carbon nanofibers, whereas, Nie et al., were studied the preparation of carbon nanofibers with antimicrobial action via embedding carbon quantum dots (CQDs) that were formerly prepared via solvothermal reaction between citric acid (CA) and 1,5-diaminonaphthalene (1,5-DAN)^25^. In another report, CQDs doped magnetic nanofibers were prepared for the self-display and removal of Hg (II) from wastewater, whereas, fluorescent CQDs were formerly prepared from PEO (polyethylene-oxide) and CS (magnetic cellulose–chitosan), under hydrothermal conditions, be homogeneously distributed within nanofibers matrix^26^. Meanwhile, such reported methods for manufacturing of biocidal/florescent nanofibers were proceeded via complicated steps as to be expressed as chemicals, energy and time consumable process.

The demonstrated approach represents unique strategy for manufacturing of fluorescent/microbicide carbon nanofibers via exploitation of PAN-nanopolymer. Whereas, PAN-nanopolymer was prepared via thermally treatment of PAN for progressed cyclization, i.e., stabilization. Successive sprout of PAN-nanopolymer was confirmed via TEM analysis. The synthesized PAN-nanopolymer was sequentially exploited in production of florescent/antimicrobial carbon nanofibers via solution blow spinning process. A comparable study between carbon nanofibers that were produced from PAN and PAN-nanopolymer were systematically demonstrated. The prepared samples were investigated via FT-IR, SEM, XRD, XPS, photoluminescence, NMR and microbicidal performance.

## Experimental work

### Materials and chemicals

Polyacrylonitrile (PAN, [C_3_H_3_N]_n_, average M_w_ = 150,000), N,N-Dimethylformamide (DMF, anhydrous, 99.8%, Sigma-Aldrich), were applied as received.

### Procedure

#### Conversion of PAN to PAN-nanopolymer

**Figure 1:**
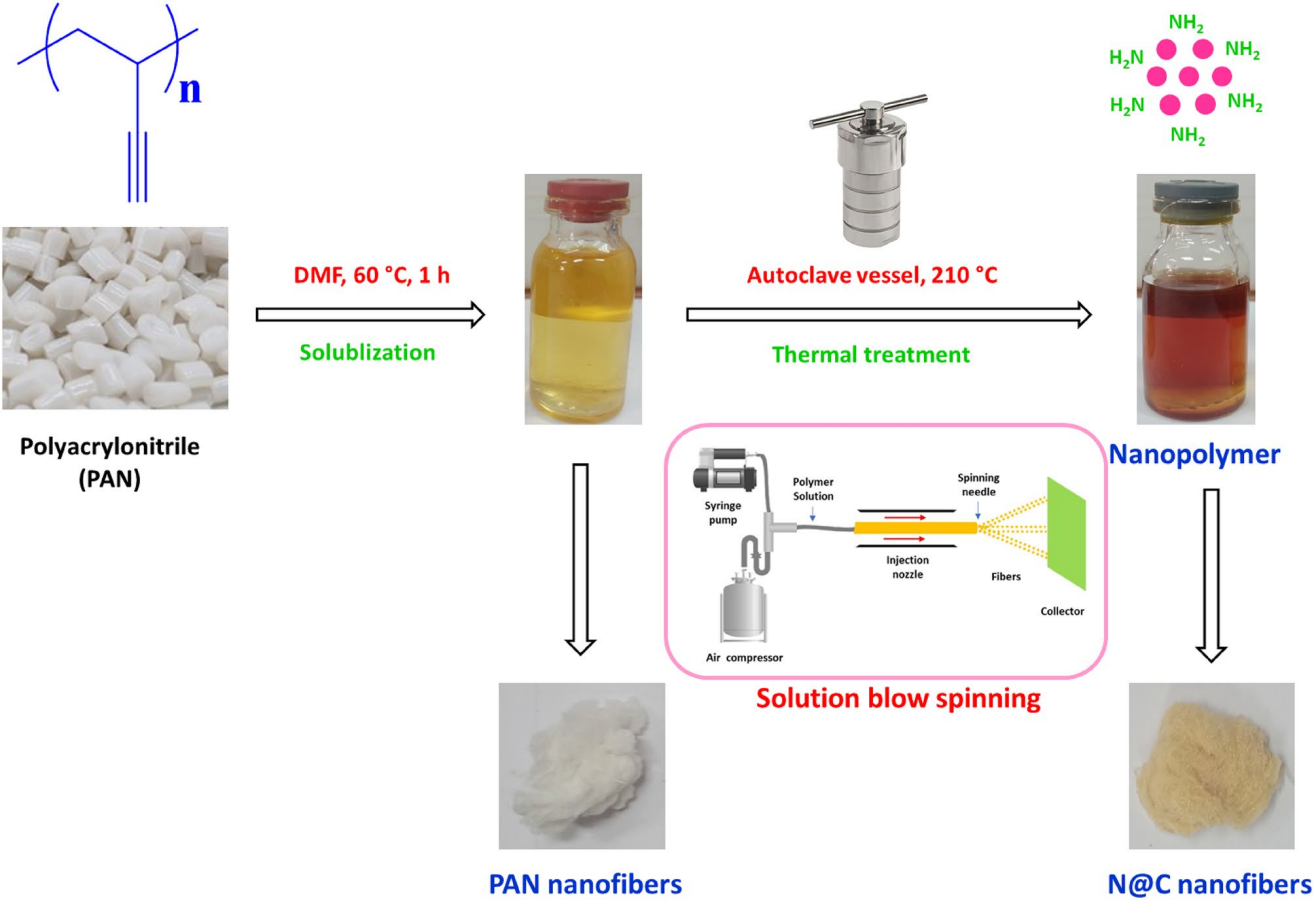
Suggested scheme for synthesis of N@C nanofibers.

According to literature^27–29^, nano-scaled polyacrylonitrile (PAN-nanopolymer) was successively clustered with the hydrothermal technique as schematically presented in Fig. 1 and according to different experimental conditions as tabulated in Table 1. Whereas, different samples were prepared with different concentrations of PAN, under the hydrothermal conditions (210 °C) for different reaction duration (2, 4 and 8 h) to give the opportunity for successive nucleation of PAN-nanopolymer, ready for spinning process.

**Table 1:** Description, physical and rheological properties of the synthesized samples.

Sample code	PAN (%, wt/vol)	Temperature (°C)	Time (h)	Form	Viscosity (mPa s)	Spin-ability
S1	10.0	210	2	Solution	501	No
S2	10.0	210	4	Solution	581	No
S3	10.0	210	8	Gel like structure	n.d.	No
S4	12.5	210	2	Honey like solution	678	Yes
S5	12.5	210	4	Honey like solution	834	Yes
S6	12.5	210	8	Gel like structure	n.d.	no
S7	15.0	210	2	Honey like solution	1210	Yes
S8	15.0	210	4	Honey like solution	1680	Yes
S9	15.0	210	8	Rubber like structure	n.d.	No
S10	20.0	210	2	Gel like structure	n.d.	No
S11	20.0	210	4	Rubber like structure	n.d.	No
S12	20.0	210	8	Rubber like structure	n.d.	No

Viscosity of blank PAN; 10.0% = 618 mPa s, 12.5% = 880 mPa s, 15% = 1130 mPa s.

#### Solution blow spinning

Solution blow spinning is a process of fiber fabrication that is proceeded via two parallel concentric streams; (i) the polymer solution that is preliminary prepared by dissolving polymer in a volatile solvent, and (ii) a pressurized gas that flows around the as-prepared polymer solution, for generation of the required nanofibers that are deposited in the direction of gas flowing. Solution-blowing device with air compressor, an injection pump, a rotary nozzle, and a fiber collector as simply shown in Fig. 1 is exploited for fiber spinning, whereas, PAN or PAN-nanopolymer solution was injected through the needle (range 16–24 gauge) at polymer flowing rate of 20–60 mL/h. Distance from the fiber collector was maintained at fifty cm from injection nozzle. Whereas, injection needle was protruded with 1 mm from concentric nozzle. The produced fibers were subsequently dried in an oven (at 60 °C) until the constant weight was achieved. The reaction duration that was required for the production of samples was 120 min and were successively obtained (25 ml/h) as a polymer flow rate. This procedure also promotes the yarn to be obtained in film or porous form.

### Characterization and instrumental analysis

High Resolution Transmission Electron Microscope (HRTEM) from Japan JEOL-JEM-1200 was manipulated for characterization of topographical and geometrical features of PAN and PAN nanopolymer, as the size distribution was estimated by “4 pi analysis software from USA” for at least 50 particles. Absorption spectral maps for PAN and PAN nanopolymer were manifested at 250–750 nm via spectroscopy “Cary 100 UV–VIS, UV–Vis–NIR Systems, from Agilent”. The florescence for the prepared PAN nanopolymer was examined via spectro-fluorometer in ultraviolet–visible range “JASCO FP8300”. The obtained data were collected at room temperature with exciting at 340 nm. Rheological properties were investigated for PAN and PAN nanopolymer through measuring the viscosity by using B-ONE Plus Viscometer from Lamy Rheology instruments.

Carbon nanofibers that were manufactured from both of PAN and PAN nanopolymer were characterized via high resolution scanning electron microscopy (HRSEM Quanta FEG 250 with field emission gun, FEI Company—Netherlands). Elemental analysis was also examined with energy dispersive X-ray analyzer, EDX (EDAX AME-TEK analyzer). Infrared spectra for carbon nanofibers were obtained from “Jasco FT/IR 6100 spectrometer”. Also, the collected absorption spectra were ranged in 4000–400 cm^−1^ (4 cm^−1^ resolution and 64 scanning times with rate of 2 mm/s). the data of both ^1^H-NMR and ^13^C-NMR were estimated from Jeol‐Ex‐300 NMR spectrometer (JEOL—Japan). Carbon nanofibers were characterized with powder X-ray diffraction using X’Pert MPD diffractometer system from Philips, at room temperature. The diffraction peaks were collected in the diffraction angle (2θ) of 3.5°–50° using monochromator (Cu Kα X-radiation at 40 kV, 50 mA and λ = 1.5418 Å).

The antimicrobial performance for the as-prepared PAN-nanopolymers and carbon nanofibers against different pathogenic strains was approved via the qualitative method of shaking flask test, whereas, the microbial reduction percent was estimated according to literature^30,31^. In this method, all of the prepared samples were examined for antimicrobial performance against three pathogenic strains of +ve gram “*Staphylococcus aurous;* ATCC-47077”, −ve gram bacterial species “*Escherichia coli;* ATCC-25922” and fungal strain “*Candida albicans;* ATCC-10231”. Briefly, the strains were aerobically cultured at 37 °C on the nutrient agar plates and inoculations were given from the fresh plates into 100 ml of LB culture medium. The bacterial growth was allowed till the optical density (OD) reached 0.1 at 550 nm, which correspond to 10^8^ colony-forming unit (CFU)/ml of cultures. Subsequently, 10 µl from above was added to 5 ml liquid NB media supplemented with 50 µg sample, and placed on Rotary Shaker (200 rpm) and incubated at 37 °C for overnight. The bacterial growth was determined by measuring optical density (OD) at 550 nm using spectrophotometer. Control broth was used without samples.

## Results and discussion

### Mechanism for preparation of carbon nanofibers based on PAN-nanopolymer

Manufacturing of nanofibers as one-dimensional structure could be ascribed as a considerable field of study as they exhibit different advantages^7^, to make it superiorly applicable in different purposes, such as in supercapacitor electrodes^8–10^, drug delivery systems^7,11^, air filters^12^, wound dressing^13^, water filters^16^, and tissue engineering^17^. The demonstrated approach represents unique strategy for manufacturing of fluorescent/microbicide carbon nanofibers via exploitation of PAN nanopolymer. Whereas, PAN nanopolymer was prepared via thermal treatment of PAN for progressed cyclization, i.e., stabilization. Figure 1 represents a suggested scheme for sprouting of PAN nanopolymer from PAN via thermal treatment at 200 °C. In accordance to literature, PAN (Tg ~ 105 °C and Tm ~ 300 °C) as one of the semicrystalline thermoplastic polymers is produced via the radical polymerization reaction, whereas, it exhibits a good resistance against different organic solvents (like; diethyl ether, chlorinated hydrocarbons, ketones and acetonitrile)^32^. When PAN is heated at high temperature (≤ 180–200 °C), the autocatalytic cyclization takes place via free radical mechanism. Whereas, such complex reaction of intra- and intermolecular cyclization (also identified as stabilization reaction), acts in preventing the thermoplastic fabrication^33^.

Furthermore, under extensive heating with autoclaving, florescent PAN nanopolymer, that are surface decorated with nitrogen containing functional groups (amino-groups), could be successively nucleated. According to literature^34^, surface decoration with such heteroatom containing functional groups resulted in acquiring the as nucleated nanopolymers a superior optical characters and biological activities. Electrospinning is a conventional technique for mass fabrication of different types of nanofibers with different morphologies^35^.

Solution blowing for fiber spinning is ascribed as an efficient/a very versatile method for synthesizing nanofiber matrix^36^, whereas, different nanofiber matrices could be successively produced to be exploited in different purposes, like to be applied as efficient membranes for air and water treatment filtration, biosensing, cell regenerating, cosmetic reagents, carriers for drug delivery, solar cells, textile industry, tissue engineering, and wound dressers^36^. Both of PAN and PAN-nanopolymer were successfully exploited for preparation of carbon nanofibers through solution blowing fiber spinning technique. Whereas, carbon nanofibers that were produced from PAN-nanopolymer are supposed to acquire optical and biological activities corresponding to its source of origination.

### Rheology properties

Different samples were prepared for examination in the current approach in order to study the effect of PAN concentration and the reaction duration for thermal treatment. According to the estimated values in Table 1, it could be declared that, PAN samples with concentration lower than 12.5% wt/vol were shown without spinnability, however, after 2 h or even 4 h of thermal treatment at 210 °C, S1 and S2 were in solution like-structure with viscosity of 501 and 581 mPa s, respectively. At concentrations of 12.5 and 15% wt/vol, honey like/spinnable samples were obtained and characterized with higher viscosity of 687 and 1210 mPa s for samples S4 and S6 that were prepared under thermal treatment for 2 h, whereas, prolonging time resulted in significant increment in the viscosity to be 834 and 1680 mPa s, for samples S5 and S7, respectively. Whereas, at the highest concentration of PAN (20% wt/vol) and regardless to the reaction duration, all the samples are non-spinnable. Eventually, it could also depict that, extending the time of thermal treatment up to 8 h, resulted in production of non-viscous/nano-spinnable samples with gel-like structures at low concentrations (10% and 12.5% wt/vol) and rubber-like structures at high concentrations of PAN (15% and 20% wt/vol). these could be attributed to the effect of thermal treatment with longer time than 4 h, could result in more complicated cyclization of PAN to be in the non-spinnable/undesirable form^33^.

### Characterization of PAN-nanopolymer

**Figure 2:**
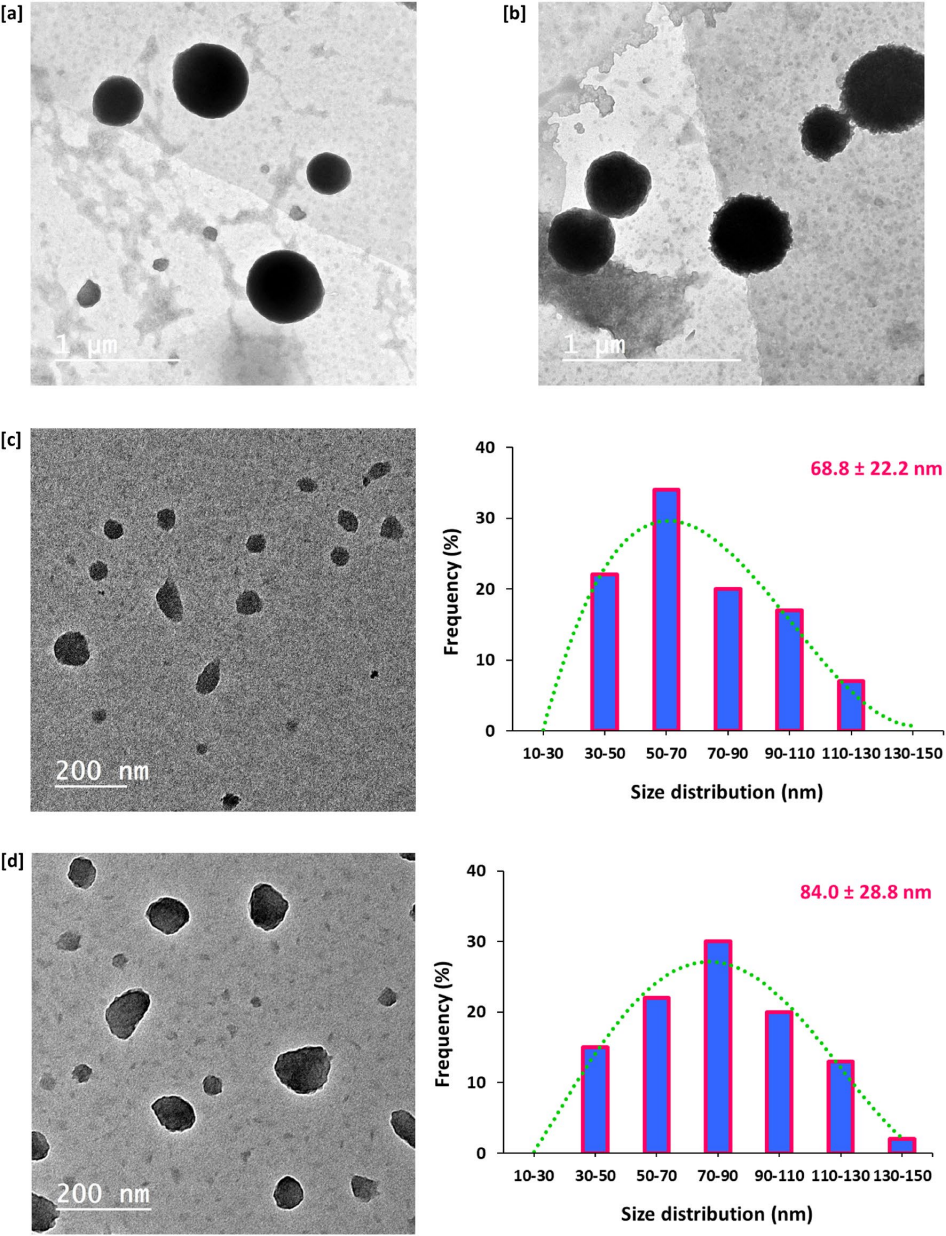
Transmission micro images (TEM) of PAN and the synthesized nanopolymer and the corresponding particle size distribution. (**a**) Blank 12.5% PAN, (**b**) Blank 15% PAN, (**c**) 12.5% PAN, 4 h and (**d**) 15% PAN, 4 h.

Topographical features and geometrical of the prepared PAN and PAN-nanopolymer could be discussed via the plotted microscopic images of Transmission Electron Microscope in Fig. 2, whereas, the size distribution was estimated and clarified for each examined sample. From the plotted data, regardless to PAN concentration, without thermal treatment, PAN macromolecules were produced in micro-size (Fig. 2a,b). Successive sprouting of spherical/nanosized PAN was performed with prolonging the reaction duration up to 4 h, whereas, the PAN sample that was prepared with 12.5% wt/vol resulted in production of PAN-nanopolymer with size distribution of 68.8 ± 22.2 nm (Fig. 2c), however, increment of concentration up to 15% wt/vol resulted in obtaining PAN-nanopolymer with size average of 84.0 ± 28.8 nm (Fig. 2d). So, it could be depicted that, the investigated technique was succeeded in tailoring PAN for nucleation of size and shape regulated PAN-nanopolymer.

**Figure 3:**
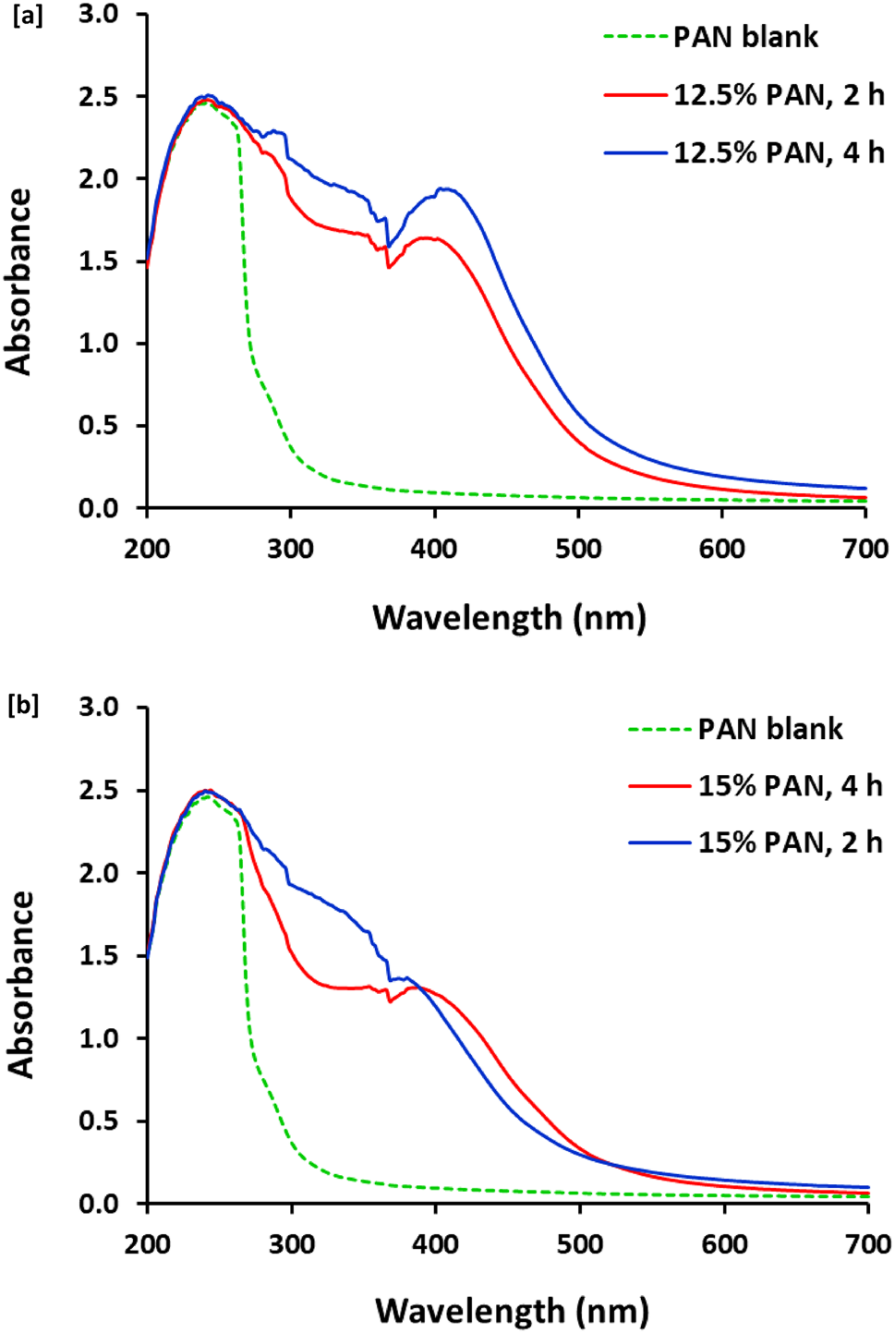
Absorbance for the synthesized nanopolymer; (**a**) 12.5% PAN and (**b**) 15% PAN.

UV–Visible spectroscopic maps are represented in Fig. 3, for PAN samples produced before and after thermal treatment with concentrations of 12.5 and 15% wt/vol, and reaction duration of 2 and 4 h. The spectral data revealed that, PAN sample that was produced without thermal treatment (blank sample) is shown with one significant band corresponding to π–π* transition (signed for nitrile group). PAN samples prepared with concentration of 12.5% with thermal treatment were observed with two characteristic peaks for π–π* transition at 250 nm (conjugation) and n–π* transition at 416 nm (electron radiation relax in a broad shoulder with long tail, for decorative/nitrogen containing groups), respectively^37,38^ (Fig. 3a). Similarly, increment of concentration up to 15% wt/vol, compared to blank sample, thermal treatment also resulted in estimation of π–π* transition peak at 250 nm and lower intense/broader n–π* transition peak with blue shifting at 390 nm, rather than samples prepared with lower concentration. Additionally, regardless to the PAN concentration, the prolongation of the reaction time for the thermal treatment resulted in slight lower intense bands. These could sign for the effect of thermal treatment in cyclization of PAN macromolecules to produce the desirable/florescent PAN-nanopolymer^37,38^.

**Figure 4:**
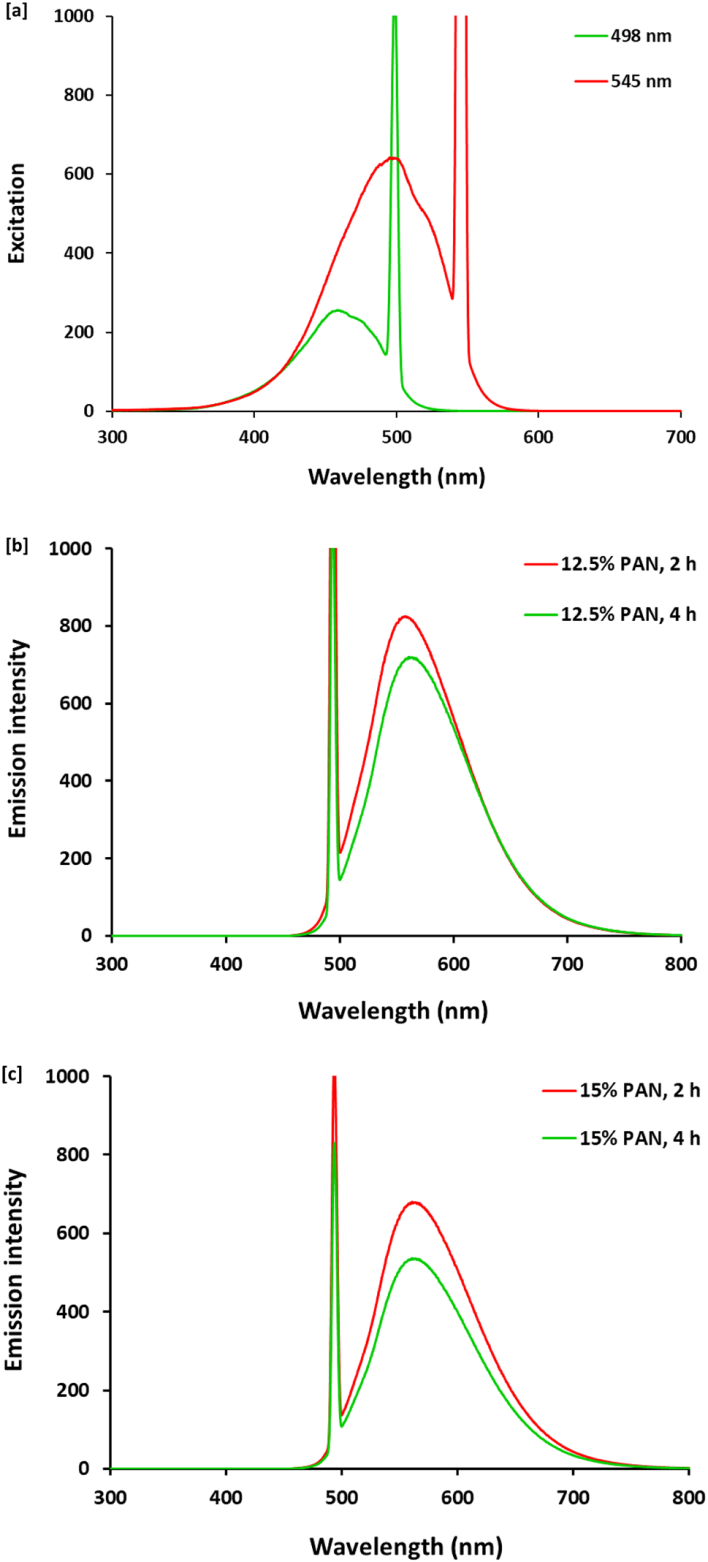
Fluorescence properties; (**a**) Excitation spectra for the synthesized nanopolymer (15% PAN, 2 h) at different wavelengths, (**b**) Emission spectra (excitation at 495 nm) for 12.5% PAN and (**c**) Emission spectra (excitation at 495 nm) for 15% PAN.

The optical activity was investigated via fluorescence emission for PAN colloids prepared with concentration of 12.5% wt/vol (Fig. 4b) and 15% wt/vol (Fig. 4c) after thermal duration to show the effect of reaction duration on the intensity of emission. Whereas, Fig. 4a represented the spectrum of emission (excitation at 498 nm and 545 nm) and from the represented data, it could be obviously observed that, (i) the emission bands in UV–Visible range can affirm the composition of PAN-nanopolymer with non-bonding and mobile pi-electrons for the decorative groups as nitrogen containing groups, (ii) longer time of thermal treatment resulted in samples with lower intense emissive peaks, attributing to the time prolongation could resulted in more complicated cyclization with lesser florescence affinity, (iii) samples prepared with lower concentration (12.5% wt/vol, Fig. 4b) were shown with higher intense emission band, that might be attributed to the higher concentrated solutions (15% wt/vol, Fig. 4c) were more sensitive for complicated cyclization with thermal treatment to produce agglomerated moieties (referring to the as-illustrated data of size distribution) with lower optical character. So, it could reveal that, the reaction duration of thermal treatment of PAN in order to produce PAN-nanopolymer is mainly affected on the intensity of emission bands, that sequentially results in the coloration of PAN-nanopolymer colloid for endowing their easy colorimetric detection. Additionally, the excitation at 498 and 545 nm resulted in estimating significant sharp emission bands in the yellow region. So, the above-illustrated data can clarify that, the nucleated PAN-nanopolymer was characterized with significant optical characters in visible light, to be superiorly applicable in production of florescent carbon nanofibers.

### Characterization of the prepared carbon nanofibers

**Figure 5:**
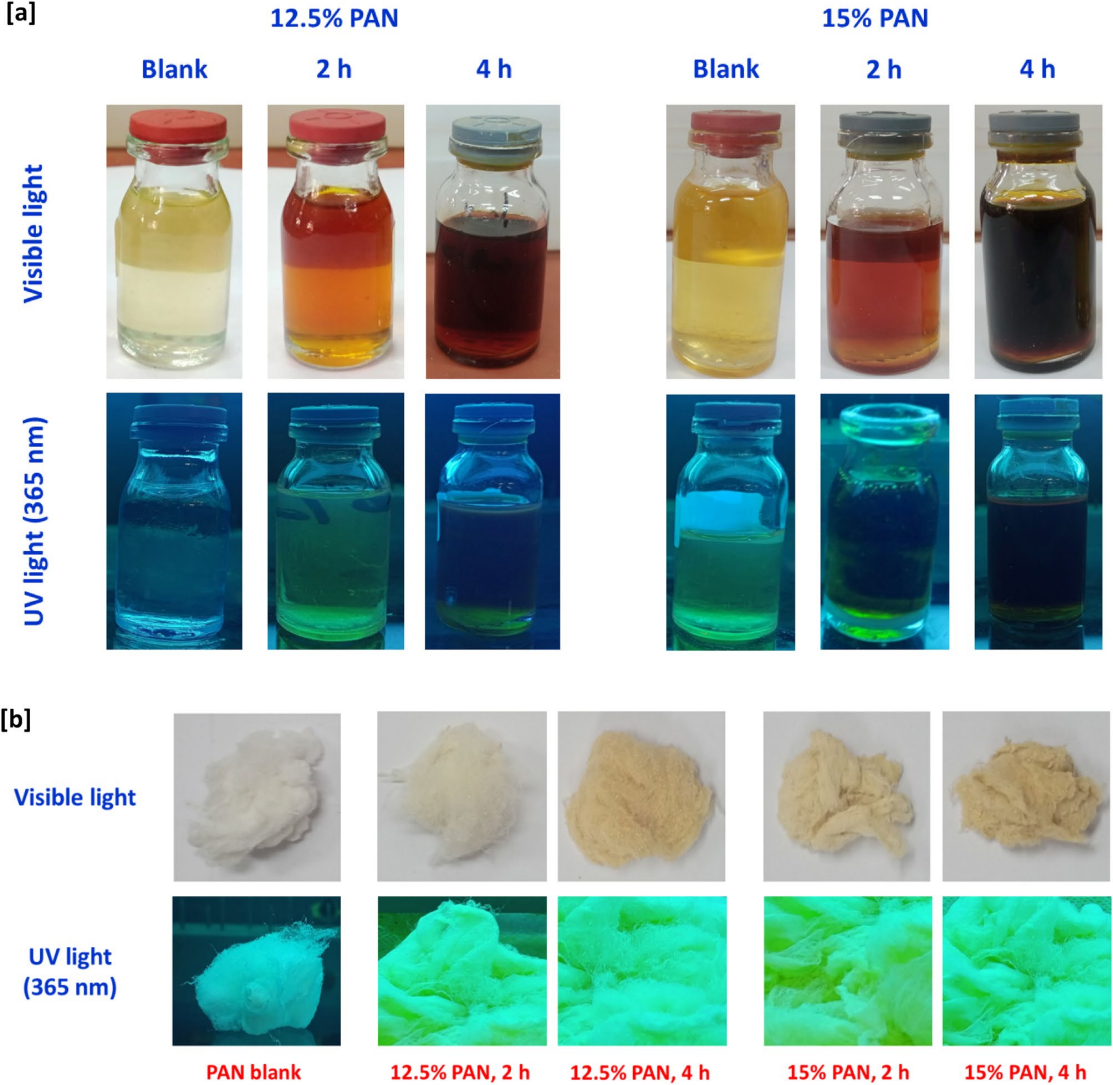
Photographical images for the synthesized nanopolymer and N@C nanofiber in the visible and UV-light (365 nm), (**a**) nanopolymer and (**b**) N@C nanofiber.

Figure 5 represents photographs that were taken in visible light and under UV-lamp for the colloidal solutions of PAN before and after thermal treatment for 2 and 4 h with two concentrations of 12.5% and 15% wt/vol (Fig. 5a), and the fibers that were prepared with exploitation of such colloids (Fig. 5b), for visual observation, to show the effect of PAN concentration and the time of thermal treatment in colour darkening. For PAN colloids, regardless to the parameter of concentration, longer time of thermal treatment resulted in darkening the colour of the prepared colloids (Fig. 5a). Similarly, fibers that obtained via the exploitation of PAN sample prepared under thermal treatment for prolonger time of 4 h is shown with more brownish colour. however, increment of PAN concentration from 12.5% wt/vol up to 15% wt/vol resulted in changing the colour of the produced fibers from creamy white to brownish colour (Fig. 5b). So, it could decide that, the visual observation for the photos in Fig. 5 is in harmony with all of the above-illustrated data, to affirm the superiority of PAN-nanopolymer in production of florescent carbon nanofibers.

**Figure 6:**
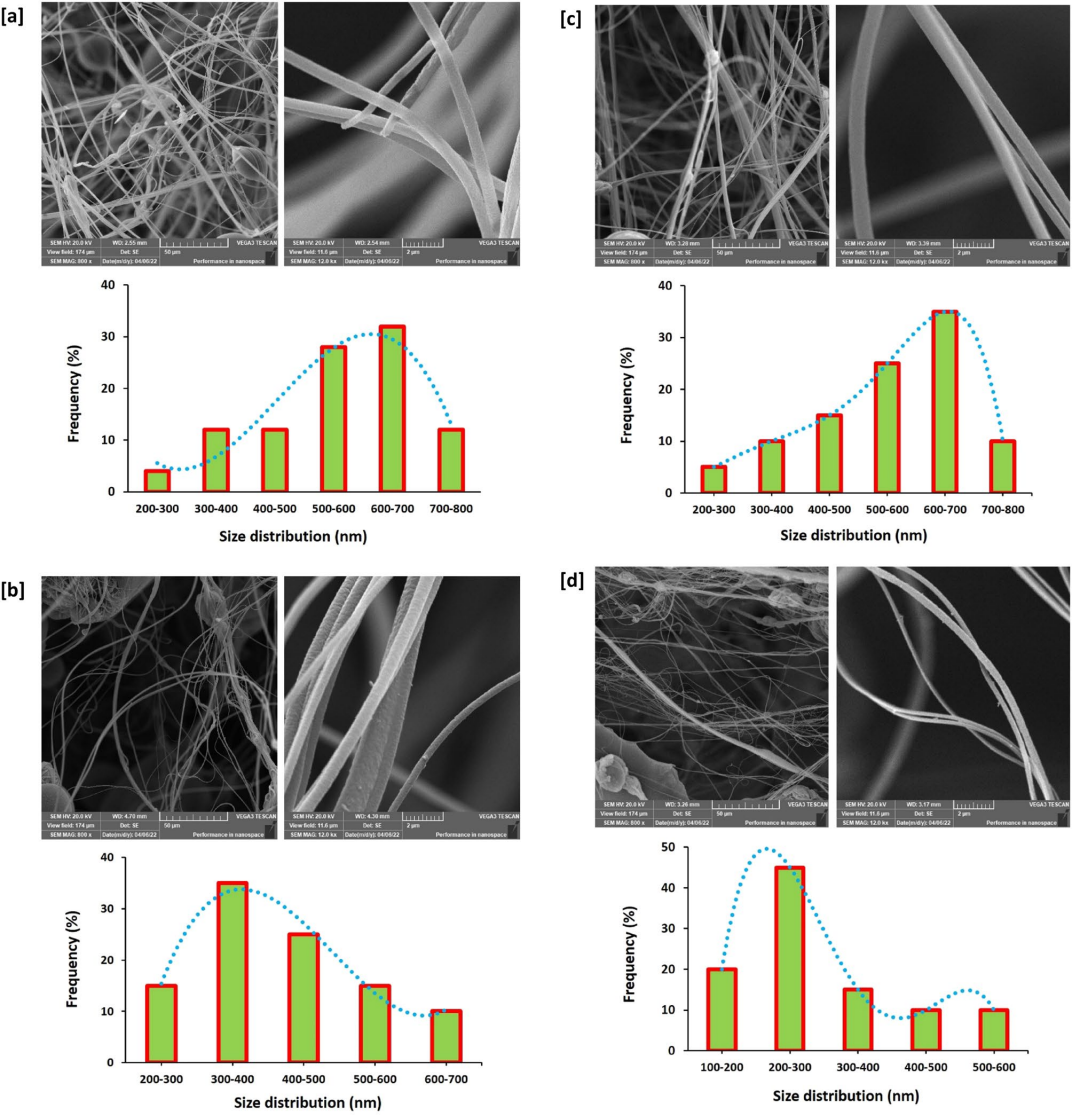

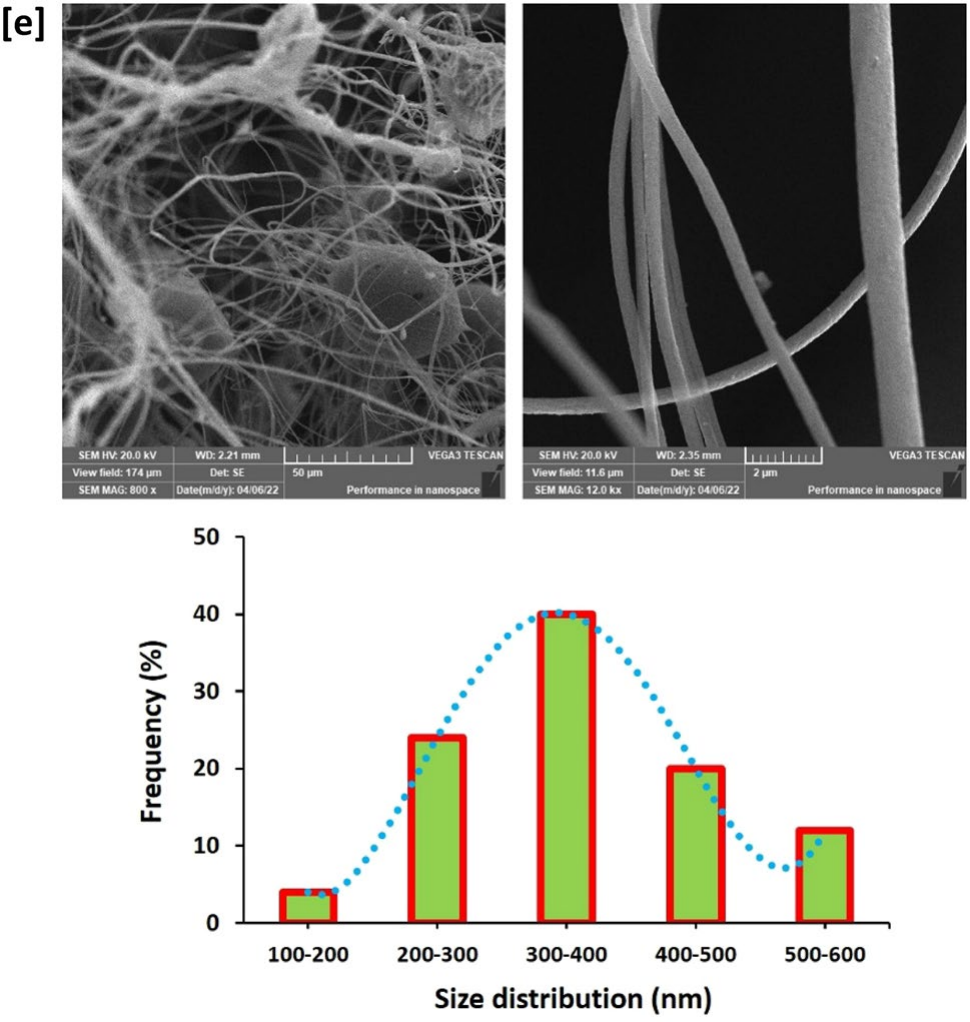
SEM images for the synthesized N@C nanofiber; (**a**) Blank PAN, (**b**) 12.5% PAN, 2 h, (**c**) 12.5% PAN, 4 h (**d**) 15% PAN, 2 h and (**e**) 15% PAN, 4 h.

Carbon nanofibers were uniquely prepared from PAN-nanopolymer. In the current approach a comparable overview is presented between the affinity of PAN versus PAN-nanopolymer for preparation of florescent/microcode carbon nanofibers. The topography of carbon nanofibers was investigated via SEM micrographs, whereas, the data are presented in Fig. 6. SEM microscopic photos showed that, carbon nanofibers prepared from PAN were exhibited with size distribution of 600–700 nm (Fig. 6a), however, exploitation of PAN-nanopolymer resulted in production of significant smaller sized carbon nanofibers. Increment of PAN-nanopolymer concentration from 12.5% wt/vol (300–400 nm, Fig. 6b) up to 15% wt/vol (200–300 nm, Fig. 6d), resulted in non-significant diminishing in the estimated value of size distribution. However, longer time of thermal treatment resulted in enlargement in size, as by comparing with the estimated value of size distribution in Fig. 6c,e; carbon nanofibers prepared from PAN-nanopolymer (12.5% wt/vol) nucleated under thermal treatment for 4 h, were analyzed with size distribution of 600–700 nm, that could be attributed to more complicated cyclization of PAN-nanopolymer under the effect of thermal treatment for longer time, that is in harmony with the previously illustrated data. The geometrical shape and size of the obtained carbon nanofibers are similar to the nanofibers prepared in literature for other polymers through electrospinning procedure^39,40^.

**Figure 7:**
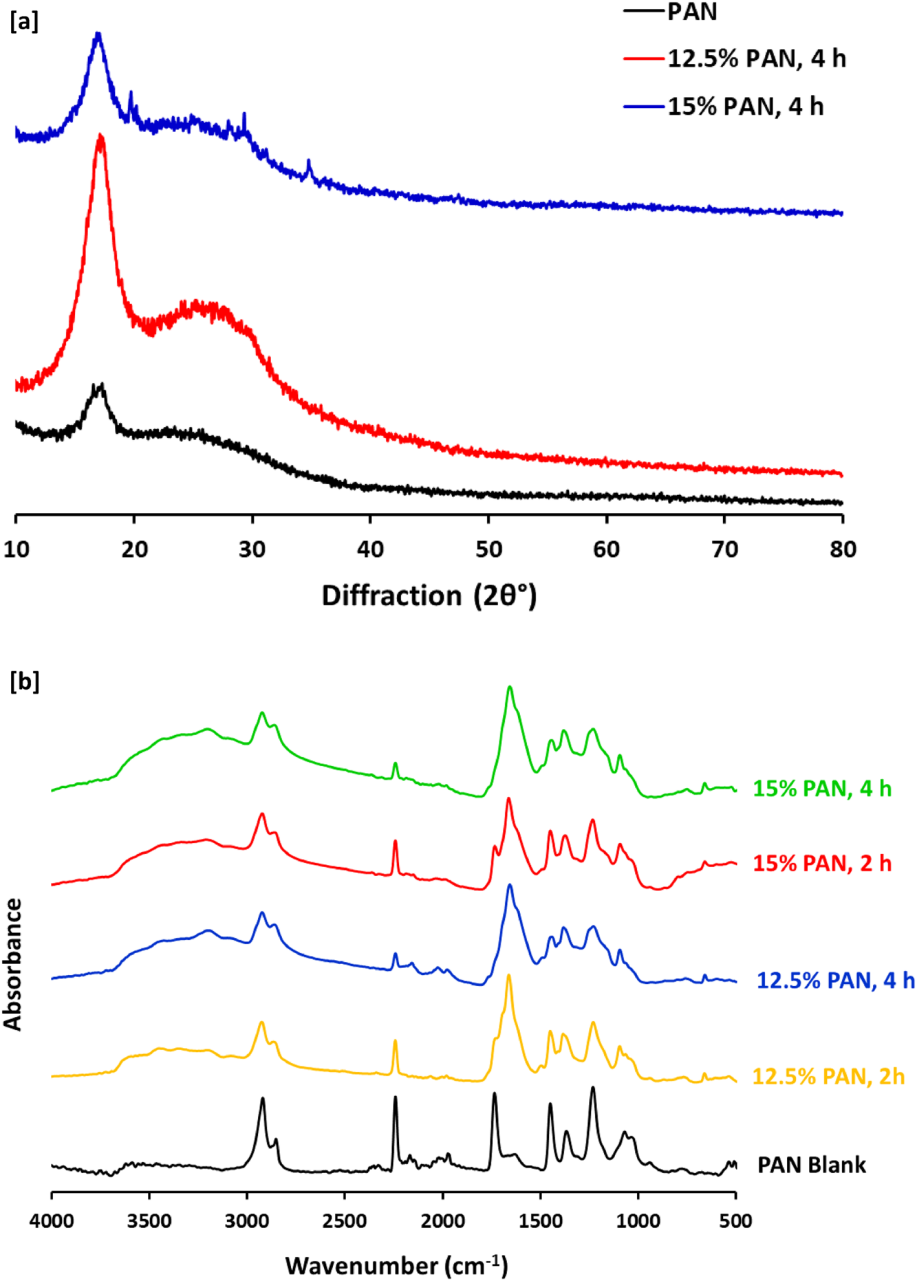
(**a**) XRD analysis and (**b**) FTIR spectra for the synthesized N@C nanofiber.

Figure 7a represents XRD analyzed data for carbon nanofibers produced from PAN & PAN-nanopolymer (12.5% and 15%), as it could be declared that, all the examined fibers were exhibited with the same diffraction peak at 2θ = 17.5°, corresponding to the (200) plane of PAN^25^, however, carbon nanofibers that were produced from lower concentrated PAN-nanopolymer (12.5% wt/vol) exhibited with an additional diffraction peak at 2θ = 26.8°, that is assigned for the crystal lattice distance of (002), while, in accordance with previous studies, this peak refers to nanosized structure with aromatic character^41,42^. Meaning that, thermal treatment of PAN with concentration of 12.5% wt/vol was succeeded in production of PAN-nanopolymer with highly regulated crystalline structure and aromatic in nature.

Figure 7b shows FTIR spectral data for carbon nanofibers produced from both of PAN and PAN-nanopolymers, and according to literature^43–46^, the plotted data could be illustrated as follows; all the examined samples were observed with the same peaks at 2846–2914 cm^−1^, 2236 cm^−1^, 1727 cm^−1^, 1445–1364 cm^−1^, 1223 cm^−1^ and 1045 cm^−1^, corresponding to sp^3^ C-H stretching, C≡N, C–N, sp^3^ C-H bending and sp^2^ C–H bending, respectively. However, the peak at 2243 cm^−1^ assigned for nitrile (C≡N) group is observed with significant lesser intensity for the analyzed carbon nanofibers produced from PAN-nanopolymers. In addition to, two new significant peaks at 1652 cm^−1^ and 1438–1363 cm^−1^ that are corresponding to C=C stretching vibration and N–H bending, respectively, for carbon nanofibers produced from PAN-nanopolymers (either 12.5% or 15% wt /vol) formerly prepared under thermal treatment for 2 h.

**Figure 8:**
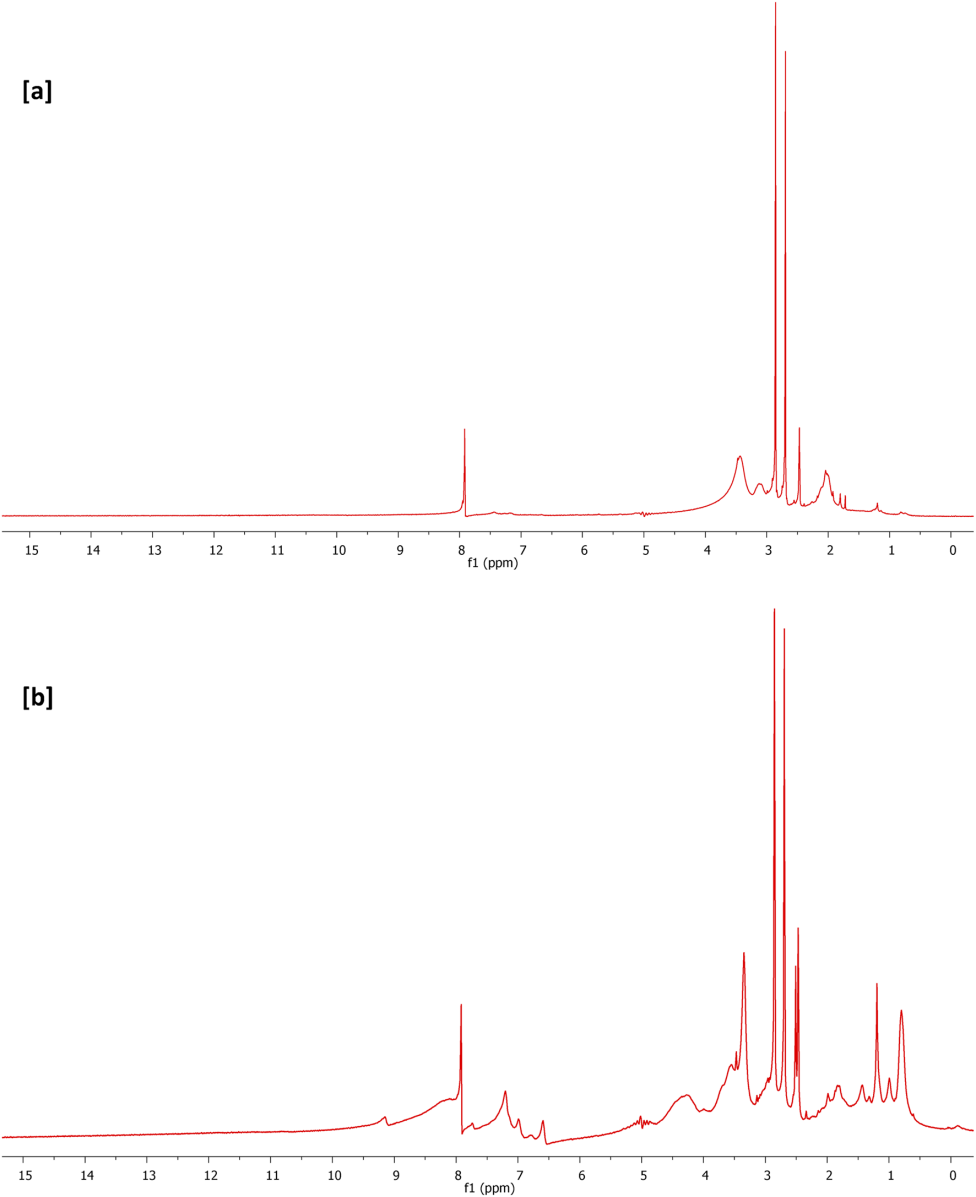

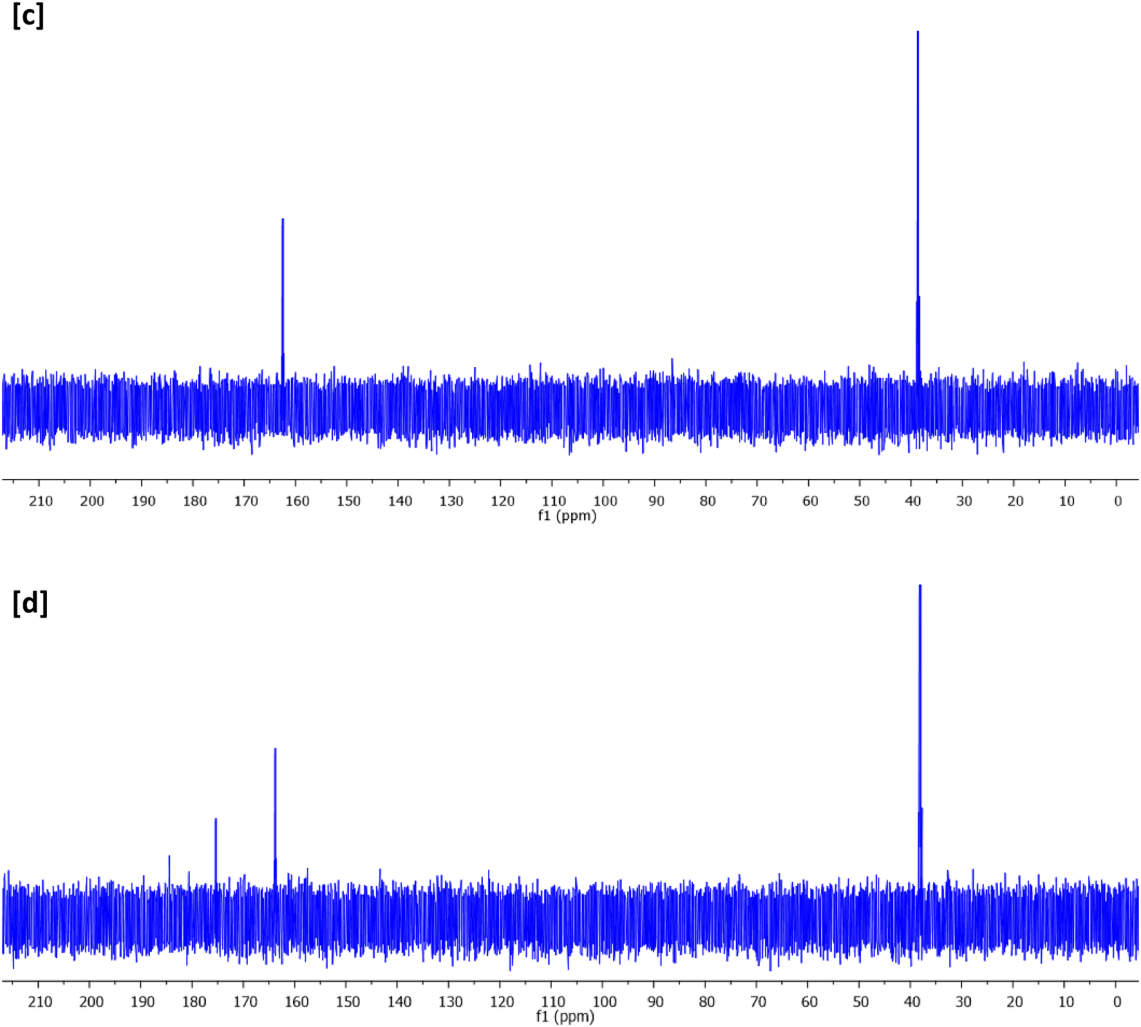
NMR spectra for the synthesized N@C nanofiber; (**a**,**b**) ^1^H-NMR, (**c**,**d**) ^13^C-NMR, (**a**,**c**) 12.5% PAN, 4 h and (**b**,**d**) 15% PAN, 4 h.

NMR spectral results are represented in Fig. 8 for confirming the chemical composition of carbon nanofibers that were formerly produced from PAN& PAN-nanopolymer. Figure 8a shows ^1^HNMR for carbon nanofibers prepared from 12.5% wt/vol PAN-nanopolymer, as it could be observed that, the characteristic bands at 1–2 ppm, 2.5–4 ppm and 8 ppm, are corresponding to the protons of sp^3^ C–H, ≡CH/–NH and protons of sp^2^ or aromatic nuclei^38,47^. Figure 8b represents ^1^HNMR spectrum of carbon nanofibers prepared from 15% wt/vol PAN-nanopolymer, so as it could be depicted that, the characteristic bands at 0.8–2 ppm, 2.5–4 ppm, 3–5 ppm, 6.5–8 ppm and 8 ppm, corresponding to protons of sp^3^ C–H, ≡CH, NH, –CH aromatic and protons of sp^2^ or aromatic nuclei^38,47^. Moreover, Fig. 8c,d represented ^13^CNMR spectra of carbon nanofibers produced from PAN-nanopolymers with two concentrations of 12.5% (Fig. 8c) and 15% (Fig. 8d) and it could be notified that, regardless to the concentration of PAN-nanopolymers, the produced carbon nanofibers are shown with characteristic bands at 35–40 ppm and 163–166 ppm, signified for sp^3^ carbons and C=C for aromatic or sp^2^ carbons, respectively^47^. All of the above-discussed affirmed the affinity of the demonstrated technique for successive nucleation of PAN-nanopolymer, whereas, the effect of thermal treatment was reflected in cyclization of PAN moieties to give PAN-nanopolymer with aromatic character, whereas, the aromatization is reflected in the as-required photoluminescent activity.

**Figure 9:**
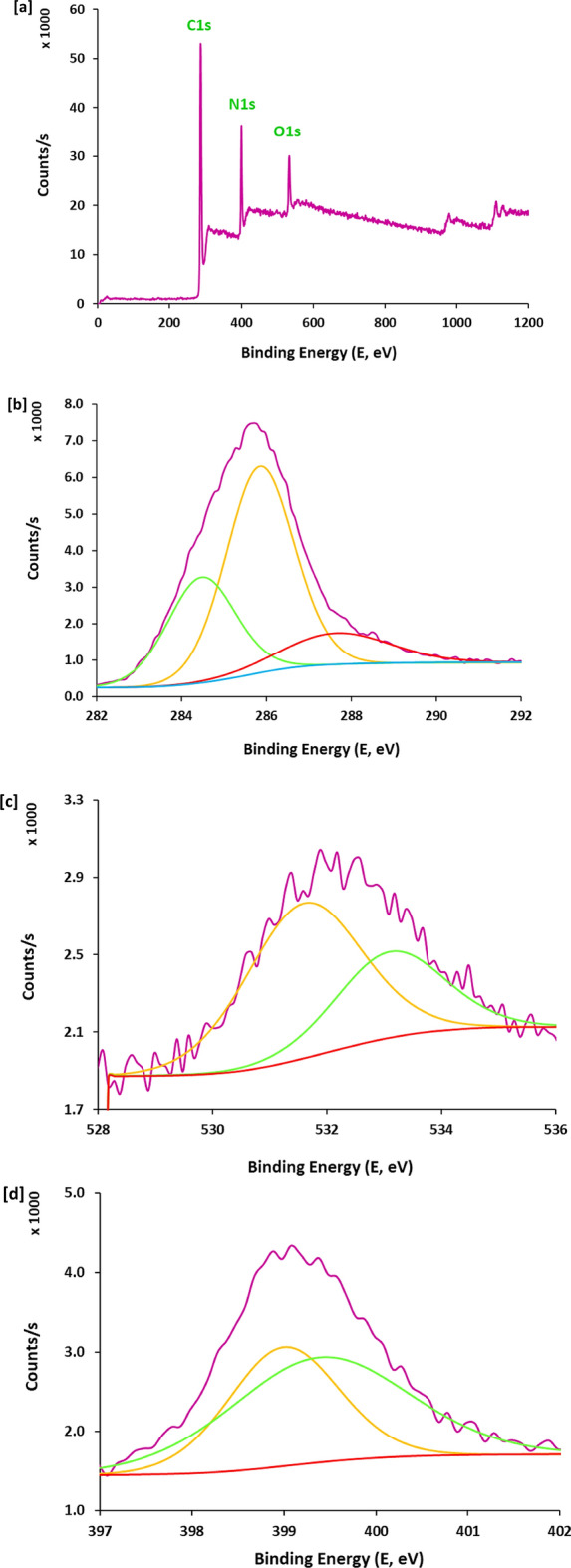
XPS spectral analysis for the synthesized N@C nanofiber (12.5% PAN, 4 h); (**a**) Survey, (**b**) C 1s, (**c**) O 1s and (**d**) N 1s.

Figure 9 represents XPS analysis for the synthesized nano-fiber (12.5% PAN, 4 h), whereas, Fig. 9a shows a survey spectrum, Fig. 9b for C 1s, Fig. 9c for O 1s and Fig. 9d for N 1s. The plotted data show that, binding energies of C1s, O1s and Ag3d, were located at 281–291, 529–535, and 364–376 eV^48^. C1s bands were estimated at binding energies of 284.1, 285.6 and 288.2 eV that were referred to C–C, C–H and C–O, respectively^49^. For O1s, binding energies peaks at 531.3 and 532.1 eV, were corresponding to C–O and C–O–O, respectively^48,50^.

### Antimicrobial performance

**Figure 10:**
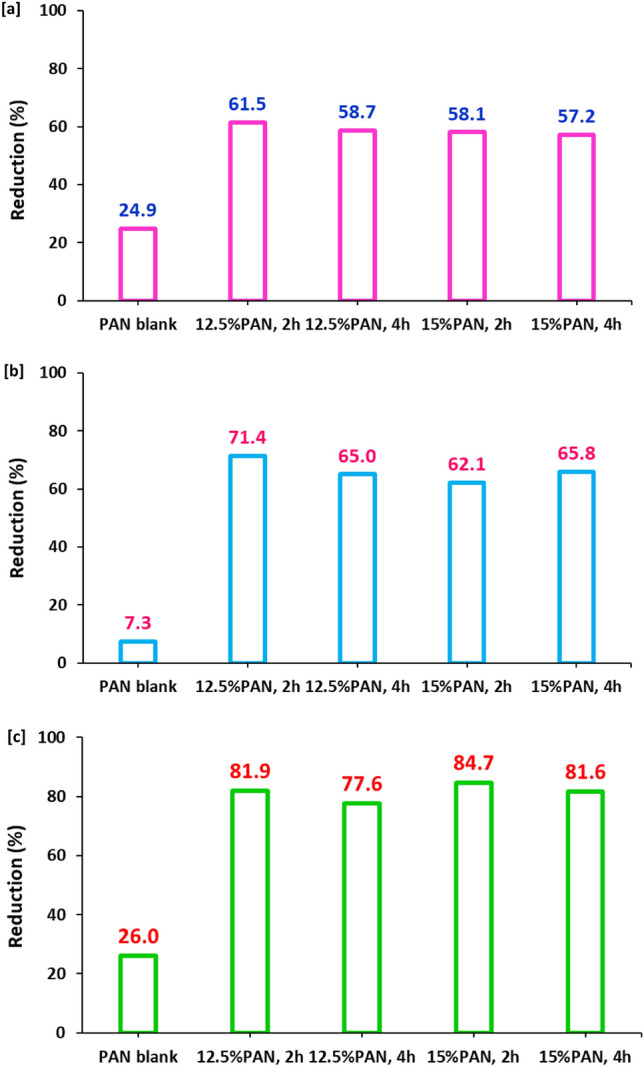
Antimicrobial activity results (bacterial reduction percentage) for the synthesized fluorescent N@C nanofiber; (**a**) St., (**b**) E. Coli and (**c**) C. Alb.

Based on literature^51–61^, the mechanism overview of antimicrobial performance for carbon nanofibers produced from aromatic characterized PAN-nanopolymer could be explained as follows; the prepared carbon nanofibers could liberate ROS in aqueous media (reactive oxygen species) i.e., oxygen and hydroxyl free radicals, that are responsible for the microbial cell death. Whereas, it could penetrate the cell wall of bacterial or fungal cells to motivate the oxidative degradation of DNA and RNA, to consequently inhibit and corrupt the genes expression. Moreover, ROS act in inactivation of the intracellular proteins, corrupts the lipids peroxidation, mitochondrial dysfunction, gradual decomposition of cell wall, and eventually followed by apoptosis, i.e., cell programmed death. In the current study, the antimicrobial performance for the produced carbon nanofibers was evaluated against three pathogens of gram-positive bacteria (*S. aureus*), gram negative bacteria (*E. coli*) and fungi (*C. albicans*) via the estimated value of microbial reduction percent (Fig. 10).

Figure 10a represents the bacterial reduction percent for *S. aureus,* as the estimated value of microbial reduction percent was 24.5%, by exploiting carbon nanofibers produced from PAN (blank), whereas, it was observably increased to 61.5%, 58.7%, 58.1% and 57.2%, in case of using carbon nanofibers produced from 12.5% PAN-nanopolymer (2 h), 12.5% PAN-nanopolymer (4 h), 15% PAN-nanopolymer (2 h) and 15% PAN-nanopolymer (4 h), respectively. However, Fig. 10b shows the bacterial reduction percent for *E. coli,* as it was 7.3%, by exploiting carbon nanofibers produced from blank, whereas, it was observably increased to 71.4%, 65%, 62.1% and 65.8%, in case of using carbon nanofibers produced from 12.5% PAN-nanopolymer (2 h), 12.5% PAN-nanopolymer (4 h), 15% PAN-nanopolymer (2 h) and 15% PAN-nanopolymer (4 h), respectively.

Moreover, in case of *C. albicans* as a fungal strain, Fig. 10c shows the fungal reduction percent*,* as the estimated value was 26%, by exploiting carbon nanofibers produced from PAN, whereas, it was superiorly increased to 81.9%, 77.6%, 84.7% and 81.6%, in case of using carbon nanofibers produced from 12.5% PAN-nanopolymer (2 h), 12.5% PAN-nanopolymer (4 h), 15% PAN-nanopolymer (2 h) and 15% PAN-nanopolymer (4 h), respectively. The plotted results obviously show that, against all the examined bacteria and fungi, carbon fibers produced from PAN-nanopolymers that formerly prepared with concentration of 12.5% under thermal treatment for 2 h, showed the highest antimicrobial performance. So, it could be summarized that, the current approach demonstrated simple/unique/time and cost saving technique for production of carbon nanofibers from PAN-nanopolymers with superior photoluminescence and antimicrobial performance.

## Conclusion

The demonstrated approach represents unique strategy for manufacturing of fluorescent/microbicide carbon nanofibers via exploitation of PAN nanopolymer. Whereas, PAN nanopolymer was prepared via thermally treatment of PAN for progressed cyclization, i.e., stabilization. Successive sprout of PAN nanopolymer was confirmed via TEM analysis. The synthesized PAN nanopolymer was sequentially exploited in production of florescent/antimicrobial carbon nanofibers. A comparable study between carbon nanofibers that were produced from PAN and PAN nanopolymer were systematically demonstrated. The prepared samples were investigated via FT-IR, SEM, XRD, XPS, photoluminescence, NMR and microbicidal performance. For limitations and future prescriptive of the current study could be mentioned compared to the reported methods in literature, whereas, the suggested process could be expressed as chemicals, energy and time saving process, to prepare carbon nanofibers with excellent performance, to be wide-scaled applicable in various biomedical and environmental purposes.

## Data Availability

The data will be available at request with Hossam Emam, hossamelemam@yahoo.com.
